# Perioperative outcomes and prognostic determinants in adult spindle cell/sclerosing rhabdomyosarcoma: implications of treatment intensity, surgical margins, and molecular heterogeneity

**DOI:** 10.3389/fonc.2026.1881225

**Published:** 2026-08-05

**Authors:** Zijing Liu, Jie Liu, Aiping Zheng, Yunfeng Tang, Yang Fu, Zeming Mo, Yaotiao Deng, Yu Jiang

**Affiliations:** Department of Medical Oncology, Cancer Center, West China Hospital, Sichuan University, Chengdu, China

**Keywords:** adult sarcoma, MYOD1 mutation, perioperative treatment, prognostic factors, spindle cell/sclerosing rhabdomyosarcoma

## Abstract

**Background:**

Spindle cell/sclerosing rhabdomyosarcoma (ssRMS) represents an uncommon and biologically heterogeneous soft tissue sarcoma with clinical outcomes in adults markedly inferior to those in pediatric populations. Perioperative treatment strategies and prognostic factors specific to adult ssRMS remain poorly defined.

**Methods:**

A total of 20 adult ssRMS patients treated at a single center were retrospectively identified between 2014 and 2023. All patients underwent surgical resection (R0/R1/R2) and received adjuvant chemotherapy (VAC or non-VAC regimens); postoperative radiotherapy was administered in 14 patients. Kaplan-Meier analysis was employed to estimate survival outcomes; log-rank testing was applied to assess between-group differences; and univariate Cox proportional hazards regression was used to calculate hazard ratios (HRs) and 95% confidence intervals.

**Results:**

Median follow-up was 35.9 months (95% CI: 28.4-43.3). Median RFS was 37.9 months (95% CI: 12.8-63.1), with 3-year RFS and OS rates of 65.0% and 74.5%, respectively. R0 resection showed a non-significant directional association with improved RFS, and postoperative radiotherapy showed a non-significant directional association with improved OS. Longer chemotherapy duration (≥12 cycles) was associated with improved OS (P = 0.030), although this finding remains exploratory because no deaths occurred in the ≥12-cycle group. Molecular tests were performed in 13 of 20 patients (65%), identifying MYOD1 mutations in 5 patients and ALK unbalanced translocations in 3 patients. Within the molecularly tested subset, progression and death events were observed only among patients harboring MYOD1 mutations or ALK unbalanced translocations.

**Conclusion:**

In this adult ssRMS cohort, multimodality perioperative treatment yielded a median RFS of 37.9 months and a 3-year OS rate of 74.5%.Surgical margins, treatment-related factors, and molecular alterations may contribute to outcome heterogeneity in adult ssRMS. MYOD1 mutations and ALK rearrangements identified subsets with more aggressive disease courses, supporting routine molecular profiling in adult ssRMS. These findings are hypothesis-generating given the small cohort size.

## 1 Introduction

Rhabdomyosarcoma (RMS) constitutes the predominant soft tissue sarcoma of childhood, comprising roughly half of all pediatric soft tissue sarcomas, yet it represents fewer than 1% of adult malignancies (1). In adults, prognosis is markedly inferior to that observed in pediatric populations, with five-year OS falling below 30% across most reported series (1). From a histological standpoint, RMS has conventionally been divided into alveolar, embryonal, and pleomorphic subtypes (1–3). Two additional variants have subsequently been recognized: spindle cell RMS, first described in 1992 as a morphologically distinct subtype with a relatively favorable prognosis in children, and sclerosing RMS, identified in 2000 and characterized by prominent hyalinization and a pseudo-vascular growth pattern (4, 5). Given their substantial histological overlap and shared molecular features, the 2013 WHO Classification of Soft Tissue Tumors merged these into a unified entity designated spindle cell/sclerosing RMS (ssRMS) (6). Importantly, whereas spindle cell RMS carries a favorable prognosis in pediatric patients, adult ssRMS behaves more aggressively, with outcomes markedly worse than in children (7, 8). Further complicating management, recurrent molecular alterations including MYOD1 hotspot mutations and ALK gene rearrangements have been identified in subsets of ssRMS; MYOD1-mutant tumors in particular are associated with high local and distant recurrence rates and disease-specific mortality exceeding 60%, underscoring the pronounced biological heterogeneity within this entity (8–10).

Perioperative management is central to the treatment of operable RMS. For the more common embryonal and alveolar subtypes, risk-stratified chemotherapy protocols have been systematically developed: the vincristine/actinomycin D (VA) and vincristine/actinomycin D/cyclophosphamide (VAC) regimens yield comparable five-year failure-free survival in low-risk patients (11); VAC/vincristine-irinotecan alternation reduces cumulative cyclophosphamide exposure without compromising efficacy in intermediate-risk disease (12); and irinotecan-containing multidrug regimens improve event-free survival in high-risk cases (13). Regarding local control, postoperative radiotherapy reduces local recurrence rates to below 15% in high-grade extremity and truncal sarcomas, though its role in retroperitoneal disease remains debated (14–17). However, these evidence-based frameworks were derived predominantly from pediatric cohorts and cannot be directly extrapolated to ssRMS, which differs in biology, molecular landscape, and clinical behavior. Whether VAC-based chemotherapy confers comparable benefit in ssRMS, and whether standard radiotherapy indications apply, remain unresolved questions.

Evidence specifically addressing the perioperative management of ssRMS is exceedingly limited, largely because this subtype is uncommon and prospective randomized evaluation remains practically unfeasible. The existing literature consists primarily of isolated case reports and small retrospective series. A 2015 retrospective study from Japan’s National Cancer Center Hospital demonstrated objective responses in 56% of ssRMS patients receiving VAC chemotherapy, suggesting its potential activity as a first-line regimen (18); however, that study included few adult patients and provided no systematic analysis of recurrence-free survival (RFS), OS, or prognostic factors. Fundamental questions therefore remain unanswered: the optimal chemotherapy regimen and treatment duration, the impact of surgical margin status on survival, and the role of postoperative radiotherapy in this specific entity. To address these gaps, this study retrospectively evaluated adult ssRMS patients managed at a single high-volume center, with the aims of characterizing RFS and OS, identifying clinical and treatment-related prognostic factors, and exploring the prognostic implications of MYOD1 mutation and ALK translocation.

## 2 Materials and methods

### 2.1 Patient eligibility

Adult patients with confirmed ssRMS who underwent treatment at West China Hospital of Sichuan University between August 2014 and March 2023 were retrospectively identified and included. Patients were eligible if they were aged 18 years or older and had undergone surgical resection for ssRMS. Patients were excluded if postoperative follow-up was three months or less, or if they had received no neoadjuvant or adjuvant therapy.

The study adhered to the ethical principles outlined in the Declaration of Helsinki, with formal approval granted by the Biomedical Research Ethics Committee of West China Hospital, Sichuan University (Approval Number: 2024-229). In view of the retrospective study design, the Committee waived the requirement for written informed consent; all data were de-identified and handled in compliance with institutional guidelines.

### 2.2 Staging and surgical assessment

Preoperative staging was performed using magnetic resonance imaging or computed tomography of the primary tumor site, supplemented by radionuclide bone scanning or 18F-fluorodeoxyglucose positron emission tomography as clinically indicated. Disease staging was performed in accordance with the Intergroup Rhabdomyosarcoma Study Group (IRSG) clinical staging system (19). Resection margin status was classified into three categories: R0 (negative margins on microscopic examination), R1 (microscopic margin involvement), or R2 (macroscopic residual disease) (20–23).

### 2.3 Pathological review and molecular characterization

All 20 cases were reviewed by the institutional soft tissue sarcoma pathology team to confirm the diagnosis of ssRMS. Diagnostic reassessment was performed in accordance with the 2020 WHO Classification of Soft Tissue and Bone Tumors (5th edition) essential diagnostic criteria to ensure consistency across the study period. No external central pathology review was performed due to the single-center retrospective study design.

Molecular testing was performed on tumor tissues obtained from surgical resection specimens or preoperative biopsy samples. Testing was performed as part of the institutional diagnostic workflow when clinically available. The mutation status of MYOD1 was assessed by Sanger sequencing. ALK unbalanced translocations and NCOA2 rearrangements were evaluated by fluorescence *in situ* hybridization (FISH). No additional molecular alterations beyond MYOD1, ALK, and NCOA2 were systematically assessed.

### 2.4 Treatment

Patients with locally advanced tumors in whom R0 resection was deemed challenging underwent preoperative biopsy, followed by neoadjuvant chemotherapy, surgical resection, and adjuvant therapy. Patients with resectable tumors proceeded directly to surgery followed by adjuvant treatment. Postoperative radiotherapy was recommended for patients with at least one established indication for adjuvant radiotherapy according to IRSG frameworks, including R1/R2 margins, nodal involvement, tumor size >5 cm, or high-grade histology (24, 25). Adjuvant chemotherapy was administered to all patients. The selection of chemotherapy regimens evolved after the publication of the 2021 guidelines from the National Comprehensive Cancer Network (NCCN) and the Chinese Society of Clinical Oncology (CSCO) for soft tissue sarcomas. Before these guidelines, in the absence of a dedicated adult-specific standard for ssRMS, chemotherapy regimens were selected based on rhabdomyosarcoma treatment principles, historical institutional practice, and physician’s discretion. After 2021 NCCN and CSCO guidelines, VAC regimen became the preferred chemotherapy for ssRMS through multidisciplinary tumor board deliberation. The number of chemotherapy cycles administered to each patient was determined by individual treatment tolerance.

### 2.5 Efficacy endpoints

RFS was measured from the date of surgery to the first recorded evidence of disease progression or death from any cause, whichever came first. OS was calculated from the date of surgery to death from any cause. Patients who experienced neither endpoint were censored at their last known follow-up date. Local recurrence was defined as disease recurrence within or contiguous to the original surgical bed or primary tumor site. Distant metastasis was defined as the development of disease at anatomic locations remote from the primary site, confirmed by imaging or histopathology. Cause of death was adjudicated by the treating multidisciplinary team and classified as disease-related (attributable to ssRMS progression or treatment-related complications) or non-disease-related (death from intercurrent illness or unrelated causes).

### 2.6 Statistical analysis

Quantitative data are reported as medians with ranges, or as frequencies and percentages. The associations between tumor location and surgical resection technique, and between surgical resection technique and postoperative radiotherapy, were evaluated using Fisher’s exact test, as the assumption of adequate expected cell counts was violated in both contingency tables. Kaplan-Meier curves were constructed to estimate survival, with between-group comparisons performed via the log-rank test. Univariate Cox proportional hazards regression was applied to derive hazard ratios and corresponding 95% confidence intervals. For subgroups with zero events, Cox regression HR estimates are unreliable and only log-rank *P* values are reported. The variables examined in survival analyses included age, gender, primary tumor location and size, preoperative IRSG stage, type of surgical resection, administration of radiotherapy, chemotherapy regimen, and number of chemotherapy cycles. Formal multivariable modeling was precluded, as the number of events was insufficient to satisfy the minimum events-per-variable criterion of 10 required for stable model estimation (RFS: 9 events; OS: 5 events); all survival analyses are therefore considered exploratory and hypothesis-generating. Tumor size data were unavailable for three patients (Cases 5, 9, and 12) owing to the absence of standardized preoperative imaging documentation. Missing tumor size values were handled by complete case analysis. Specifically, these three patients were retained in all survival analyses not involving tumor size as a stratification variable and were excluded only from the tumor size subgroup comparison in univariate analysis. Given that the proportion of missing data was limited and that this missingness was confined to a single covariate, formal multiple imputation was not performed. Given the inclusion of both upfront-surgery and neoadjuvant-chemotherapy patients, an exploratory subgroup analysis was performed to compare survival between the two initial treatment strategies using log-rank testing, and univariable Cox regression. In addition, all primary Kaplan-Meier and univariable Cox analyses were repeated in the upfront-surgery subgroup (Cases 1–17) to evaluate the robustness of the principal findings. All statistical analyses were performed using SPSS version 26.0 (IBM Corp, Armonk, NY, USA), R version 4.5.2 (R Foundation for Statistical Computing, Vienna, Austria), and GraphPad Prism 8.0.1 (GraphPad Software, San Diego, CA, USA). Statistical significance was defined as a two-sided *P*-value below 0.05. Findings from subgroups with zero events are considered exploratory regardless of the *P*-value threshold.

## 3 Results

### 3.1 Patients characteristics

Between August 2014 and March 2023, 22 adult patients diagnosed with ssRMS underwent surgical resection. A total of two patients were removed from the analysis: one owing to inadequate follow-up documentation, and one who received neither preoperative nor postoperative adjuvant therapy. A total of 20 patients were included in the final analysis (15 males and 5 females), with a median age of 31.5 years (range: 19-66 years). Primary tumor sites included the head and neck (n = 10), limbs (n = 6), genitourinary tract (n = 2), and trunk (n = 2). Pathologically, 15 cases were classified as spindle cell type, 3 as sclerosing type, and 2 as mixed spindle cell/sclerosing type. Molecular testing revealed MYOD1 mutations in 5 patients and unbalanced ALK gene translocations in 3 patients. Detailed clinicopathological features are presented in **Table 1**.

**Table 1:** Clinical and pathological characteristics of 20 adult patients with spindle cell/sclerosing rhabdomyosarcoma.

Case	Age	Sex	Primary site	Pathological subtype	Genetic mutations	Size of the primary tumor	Nodal involvement	Stage	Group classification
1	42	Male	Vocal cords	Spindle Cell	Not tested	≤5cm	Negative	1	IIa
2	29	Female	Right lower leg	Spindle Cell/Sclerosing	Not tested	≤5cm	Positive	3	IIb
3	56	Male	Right eye orbital tissue	Spindle Cell	Not tested	≤5cm	Negative	1	IIIb
4	27	Male	Left mandible	Spindle Cell	Unbalanced translocation of the ALK gene	≤5cm	Negative	1	IIa
5	32	Male	Prostate	Spindle Cell	Not tested	Unknown	Negative	2	Ia
6	34	Female	Hypopharynx	Sclerosing	MYOD1 wild-type; NCOA2 negative	≤5cm	Negative	1	IIa
7	54	Female	Right foot	Spindle Cell	Not tested	>5cm	Negative	3	Ib
8	66	Male	Right shoulder	Spindle Cell	MYOD1 wild-type; NCOA2 negative	≤5cm	Negative	2	Ia
9	25	Male	Left cervical region	Spindle Cell	MYOD1 mutation	Unknown	Negative	1	IIIb
10	19	Male	Proximal left tibia	Spindle Cell	MYOD1 wild-type	>5cm	Positive	3	IIb
11	31	Male	Right floor of mouth	Spindle Cell	MYOD1 wild-type; NCOA2 negative	≤5cm	Negative	1	Ia
12	22	Female	Dorsum of the tongue	Spindle Cell	Unbalanced translocation of the ALK gene; NCOA2 negative	Unknown	Negative	1	Ia
13	20	Male	Right mandible	Spindle Cell	MYOD1 mutation; NCOA2 negative	≤5cm	Negative	1	Ia
14	51	Female	Right mandible	Spindle Cell	Unbalanced translocation of the ALK gene	≤5cm	Negative	1	IIa
15	49	Male	Right buccal region	Spindle Cell	MYOD1 mutation	≤5cm	Negative	1	Ia
16	33	Male	Left upper limb	Sclerosing	Not tested	≤5cm	Negative	2	IIIb
17	21	Male	Right testis	Sclerosing	Not tested	≤5cm	Positive	1	IIb
18	28	Male	Radial side of the left forearm	Spindle Cell	MYOD1 mutation	>5cm	Negative	3	Ib
19	24	Male	Right pelvis	Spindle Cell/Sclerosing	MYOD1 mutation	>5cm	Negative	3	IIa
20	52	Male	Left lower leg	Spindle Cell	NCOA2 negative	≤5cm	Negative	2	Ia

“Unknown” in the tumor size column indicates that standardized preoperative cross-sectional imaging was unavailable for these patients.

### 3.2 Treatment characteristics of operable patients

All 20 patients underwent surgical resection, including 17 who proceeded directly to surgery and 3 who received neoadjuvant chemotherapy prior to resection following preoperative biopsy confirmation. Margin status was R0 in 12 patients, R1 in 5, and R2 in 3. No significant association was found between tumor location and resection margin status (Fisher’s exact test, *P* = 0.325).

All patients received adjuvant chemotherapy following surgery. Twelve patients received VAC-based regimens for 5-16 cycles, while 8 received non-VAC regimens for 3-12 cycles. Of the 10 patients with head and neck primaries, 8 received VAC and 2 received AI-based regimens. Of the 6 patients with extremity primaries, 2 received VAC, 3 received AI-based regimens, and 1 received VAI. By the stage, 8 of 11 stage 1 patients (72.7%) received VAC, 3 of 4 stage 2 patients (75.0%) received VAC, and only 1 of 5 stage 3 patients (20.0%) received VAC, with the 3 stage 3 patients receiving AI-based.

All 20 patients met at least one IRSG-based indication for adjuvant radiotherapy. However, 14 patients received radiotherapy, whereas 6 patients declined treatment for personal reasons, with no documented contraindications. Among the treated patients, 13 received conventional X-ray fractionated irradiation (59.4-66.0 Gy) and 1 received heavy particle radiotherapy. Although patients with non-R0 resections tended to receive radiotherapy more frequently, this association did not reach statistical significance (Fisher’s exact test, *P* = 0.065). Treatment details and individual patient outcomes are summarized in **Table 2**.

**Table 2:** Perioperative treatment and corresponding prognosis in adult patients with spindle cell/sclerosing rhabdomyosarcoma.

Case	Types of resection	Chemotherapy	Postoperative radiotherapy	Progress (months)	Pattern of progression	Site of metastasis	Cause of death	Survival (months)
1	R1	12-cycle adjuvant VAC	60.2Gy (2.15Gy/f, 1f/d)	17.3	Local recurrence	–	–	Alive at 56.3
2	R0	12-cycle adjuvant VAC	60.2Gy (2.15Gy/f, 1f/d)	No	None	–	–	Alive at 83.1
3	R2	6-cycle adjuvant AI	60Gy (2Gy/f, 1f/d)	No	None	–	–	Alive at 48.4
4	R1	2-cycle AI→1-cycle adjuvant VDC	60Gy (2Gy/f, 1f/d)	7.1	Local recurrence and distant metastasis	Sternum	Disease-related	Died at 16.4
5	R0	10-cycle adjuvant VAC	No	15.4	Local recurrence	–	Disease-related	Died at 25.9
6	R1	14-cycle adjuvant VAC	Dose unknown, 28f	No	None	–	–	Alive at 38.6
7	R0	4-cycle adjuvant AI	No	16.1	Distant metastasis	Right para-external iliac vascular region, right inguinal region and right lower extremity intermuscular space	Disease-related	Died at 30.3
8	R0	14-cycle adjuvant VAC	60Gy (2Gy/f, 1f/d)	No	None	–	–	Alive at 35.9
9	R2	5-cycle adjuvant VAC and VI alternating	60Gy (2Gy/f, 1f/d)	10.0	Local recurrence	–	Disease-related	Died at 30.9
10	R0	3-cycle adjuvant AI	No	No	None	–	–	Alive at 32.4
11	R0	16-cycle adjuvant VAC	60Gy (2Gy/f, 1f/d)	No	None	–	–	Alive at 29.2
12	R0	8-cycle adjuvant VAC	60.2Gy (2.15Gy/f, 1f/d)	No	None	–	–	Alive at 19.5
13	R0	12-cycle adjuvant VAC	66Gy (2.2Gy/f, 1f/d)	No	None	–	–	Alive at 26.9
14	R1	7-cycle adjuvant VAC	No	12.2	Distant metastasis	Bilateral lungs	–	Alive at 26.0
15	R0	9-cycle adjuvant VAC	59.4Gy (1.8Gy/f, 1f/d)	No	None	–	–	Alive at 29.9
16	R2	12-cycle adjuvant VAC	59.4Gy (1.8Gy/f, 1f/d)	18.6	Local recurrence	–	–	Alive at 35.7
17	R0	6-cycle adjuvant TC	60Gy (2Gy/f, 1f/d)	No progression at 85.3	None	–	–	Alive at 122.6
18	R0	2-cycle neoadjuvant AI→10-cycle adjuvant VDC and IE alternating	60Gy (2Gy/f, 1f/d)	37.9	Local recurrence	–	–	Alive at 58.6
19	R1	3-cycle neoadjuvant IAP→8-cycle adjuvant IAP	No	36.2	Local recurrence and distant metastasis	Right forearm, right lung, right psoas major muscle, erector spinae muscle and left lower leg	Disease-related	Died at 47.7
20	R0	2-cycle neoadjuvant VAI→4-cycle adjuvant VAI	No	No	None	–	–	Alive at 24.1

None, no disease progression detected during follow-up; –, not applicable.

VAC, vincristine, actinomycin D, and cyclophosphamide; AI, doxorubicin and ifosfamide; VDC, vincristine, doxorubicin, and cyclophosphamide; VI, vincristine and irinotecan; TC, paclitaxel + carboplatin; IE, ifosfamide + etoposide; IAP, doxorubicin, cisplatin, and ifosfamide; VAI, vincristine, actinomycin D, and ifosfamide.

### 3.3 Efficacy of postoperative adjuvant chemoradiotherapy

**Figure 1:**
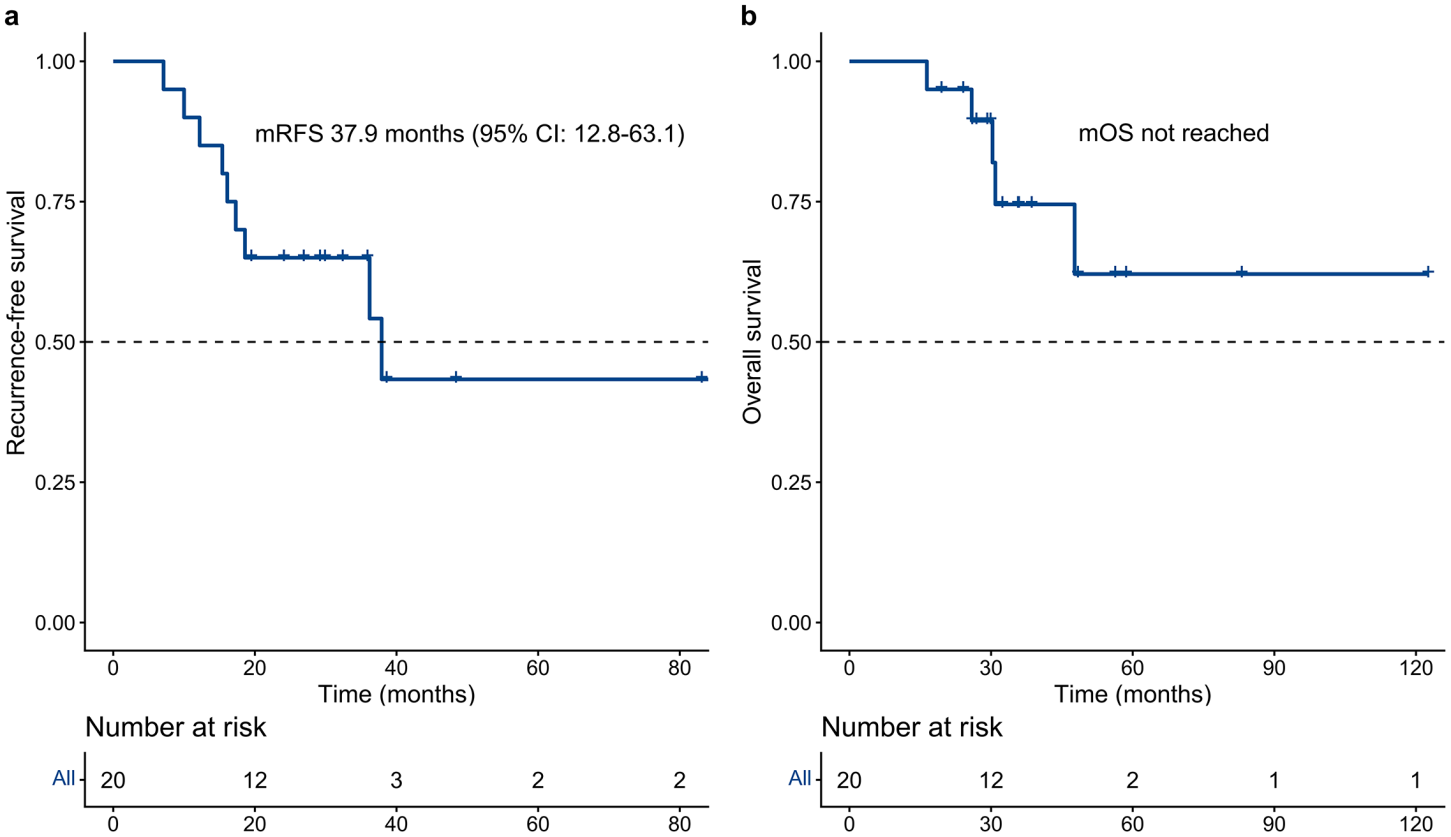
Kaplan-Meier curves of recurrence-free survival **(a)** and overall survival **(b)** for all patients.

The data cutoff was November 12, 2024, with a median follow-up of 35.9 months (95% CI: 28.4-43.3). At the end of follow-up, 9 patients had experienced disease progression. An mRFS of 37.9 months (95% CI: 12.8-63.1) was observed, with RFS rates at 1, 2, and 3 years of 90.0%, 65.0%, and 65.0%, respectively (**Figure 1a**). Five patients died of disease during follow-up and the remaining 15 were alive at last follow-up. The mOS was not reached over the follow-up period, with OS rates at 1, 2, and 3 years of 100%, 95.0%, and 74.5%, respectively (**Figure 1b**).

Among the 9 patients who experienced disease progression, local recurrence alone was the predominant pattern, occurring in 5 patients (55.6%), while isolated distant metastasis was identified in 2 patients (22.2%), and concurrent local recurrence with distant metastasis was observed in the remaining 2 patients (22.2%). All 5 deaths that occurred during the follow-up period were attributable to disease progression, with no non-disease-related mortality recorded in this cohort. Detailed patterns of progression and causes of death for individual patients are provided in **Table 2**.

### 3.4 Prognostic factors affecting RFS and OS

**Figure 2:**
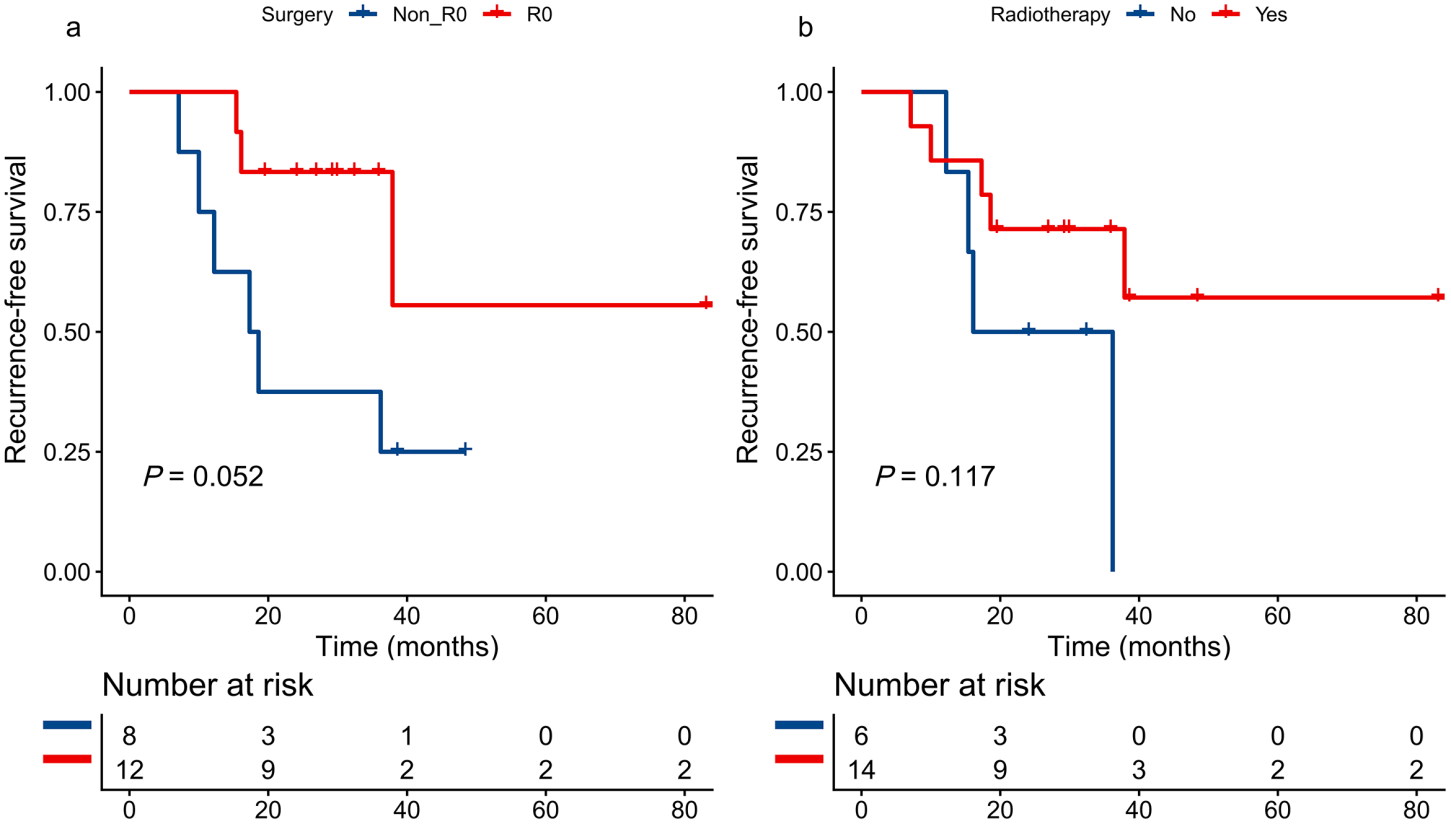
Univariate analyses of recurrence-free survival stratified by resection margin status (R0 vs. non-R0) **(a)** and postoperative radiotherapy **(b)**.

In univariate analysis, R0 resection showed a non-significant directional association with improved RFS compared with non-R0 resection (HR 0.27, 95% CI 0.07-1.10; log-rank *P* = 0.052; **Figure 2a**). Patients who received postoperative radiotherapy also demonstrated a non-significant directional association with improved RFS than those who did not (mRFS: not reached vs. 16.1 months; HR 0.34, 95% CI 0.08-1.39; log-rank *P* = 0.117; **Figure 2b**). Complete univariate RFS results are provided in **Table 3**.

**Table 3:** Univariate analysis of recurrence-free survival in all patients.

Variable	No. of events/patients	Univariable	
		Adjusted HR (95% CI)	*P*-value (log-rank)
Gender			
Male	7/15	1.30 (0.27, 6.32)	0.748
Female	2/5	1	
Age			
≤30	4/9	1	0.816
>30	5/11	1.17 (0.31, 4.45)	
Primary site			
Extremity	3/6	1	0.943
Other sites	6/14	1.05 (0.26, 4.22)	
Tumor size			
≤5cm	4/13	0.43 (0.10, 1.96)	0.264
>5cm	3/4	1	
Unknown	2/3	–	
Preoperative IRSG staging			
1	4/11	1	0.649
2-3	5/9	1.36 (0.36, 5.07)	
Surgery			
R0 resection	3/12	0.27 (0.07, 1.10)	0.052
Non-R0 resection	6/8	1	
Radiotherapy			
Yes	5/14	0.34 (0.08, 1.39)	0.117
No	4/6	1	
Chemotherapy regimen			
VAC	5/12	1.05 (0.27, 4.01)	0.945
Non-VAC	4/8	1	
Number of chemotherapy cycles			
≥12	3/8	0.53 (0.13, 2.12)	0.358
<12	6/12	1	
Initial treatment strategy			
Upfront surgery	7/17	0.86 (0.18, 4.22)	0.857
Neoadjuvant chemotherapy + surgery	2/3	1	

HR and 95% CI were estimated using univariate Cox proportional hazards regression. *P*-values are derived from the log-rank test.

The dash (–) in the “Unknown” tumor size row indicates that hazard ratio estimation was not applicable, as these three patients were excluded from the tumor size subgroup analysis due to unavailable preoperative imaging data. They were included in all other univariate analyses presented in this table.

IRSG, Intergroup Rhabdomyosarcoma Study Group; HR, hazard ratios; CI, confidence intervals.

**Figure 3:**
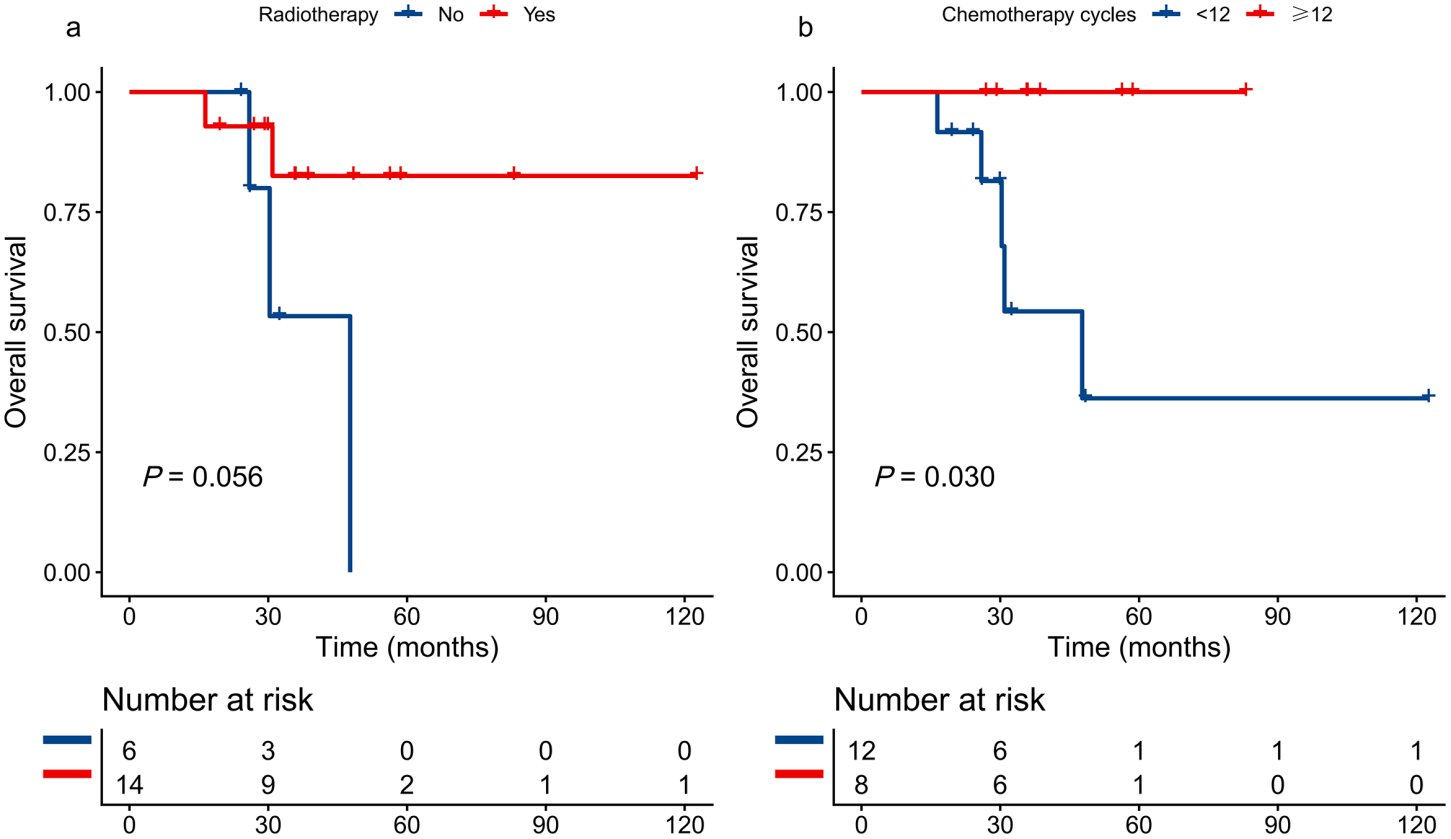
Univariate analyses of overall survival stratified by postoperative radiotherapy **(a)** and number of chemotherapy cycles (<12 vs. ≥12) **(b)**.

Regarding OS, patients who received radiotherapy showed a non-significant directional association with improved survival over those who did not (mOS: not reached vs. 47.7 months; HR 0.20, 95% CI 0.03-1.23; log-rank *P* = 0.056; **Figure 3a**). Of note, patients receiving ≥12 cycles demonstrated significantly better OS than those receiving <12 cycles (log-rank *P* = 0.030; **Figure 3b**). Complete univariate OS results are provided in **Table 4**.

**Table 4:** Univariate analysis of overall survival in all patients.

Variable	No. of events/patients	Univariable	
		Adjusted HR (95% CI)	*P*-value (log-rank)
Gender			
Male	4/15	1	0.927
Female	1/5	0.90 (0.10, 8.14)	
Age			
≤30	3/9	1	0.698
>30	2/11	0.70 (0.11, 4.30)	
Primary site			
Extremity	1/6	0.48 (0.05, 4.32)	0.503
Other sites	4/14	1	
Tumor size			
≤5cm	1/13	0.22 (0.02, 2.47)	0.180
>5cm	2/4	1	
Unknown	2/3	–	
Preoperative IRSG staging			
1	2/11	0.69 (0.12, 4.17)	0.685
2-3	3/9	1	
Surgery			
R0 resection	2/12	0.59 (0.10, 3.58)	0.562
Non-R0 resection	3/8	1	
Radiotherapy			
Yes	2/14	0.20 (0.03, 1.23)	0.056
No	3/6	1	
Chemotherapy regimen			
VAC	2/12	0.59 (0.10, 3.66)	0.566
Non-VAC	3/8	1	
Number of chemotherapy cycles			
≥12	0/8	–†	0.030
<12	5/12	1	
Initial treatment strategy			
Upfront surgery	4/17	0.94 (0.10, 8.73)	0.953
Neoadjuvant chemotherapy + surgery	1/3	1	

†Because no death events occurred in the ≥12 chemotherapy cycles group during follow-up, the Cox regression hazard ratio estimate is unreliable. Accordingly, only the log-rank P value is reported here.

HR and 95% CI were estimated using univariate Cox proportional hazards regression. *P*-values are derived from the log-rank test.

The dash (–) in the “Unknown” tumor size row indicates that hazard ratio estimation was not applicable, as these three patients were excluded from the tumor size subgroup analysis due to unavailable preoperative imaging data. They were included in all other univariate analyses presented in this table.

IRSG, Intergroup Rhabdomyosarcoma Study Group; HR, hazard ratios; CI, confidence intervals.

Because the cohort included different initial treatment strategies, an exploratory comparison was performed. No significant differences were observed in RFS (log-rank *P* = 0.857) or OS (log-rank *P* = 0.953). However, given the very small neoadjuvant subgroup (n = 3), these findings should be interpreted with caution.

### 3.5 Effect of MYOD1 gene mutation and ALK gene unbalanced translocation on patient prognosis

Among the 20 patients, 13 (65%) underwent at least one molecular assay. Detailed per-patient testing profiles are provided in **Table 1**. MYOD1 mutational status was assessed in 9 patients, of whom 5 (55.6%) harbored MYOD1 mutations and 4 (44.4%) were wild-type. ALK FISH was performed in 3 patients, all of whom showed ALK unbalanced translocations. NCOA2 gene translocations were not detected in any of the 6 patients. The remaining 7 patients received no molecular testing: 5 were diagnosed before systematic molecular profiling was integrated into the institutional diagnostic workflow, and 2 declined testing owing to personal or financial considerations. No patient harbored both MYOD1 mutation and ALK translocations concurrently.

Among the 5 MYOD1-mutant patients, 3 (60.0%) experienced disease progression and 2 (40.0%) died during follow-up. In contrast, none of the 4 MYOD1 wild-type patients experienced disease progression or death over a follow-up of 29.2–38.6 months. Among the 3 patients with ALK unbalanced translocations, 2 (66.7%) experienced disease progression and 1 (33.3%) died. Given the limited number of patients with available molecular data, these findings are presented as exploratory observations.

### 3.6 Subgroup analysis of patients undergoing upfront surgery

To evaluate the robustness of the primary findings within the more homogeneous upfront-surgery population, all primary survival analyses were repeated in the 17 patients who underwent upfront surgery. In this restricted cohort, median RFS was not reached, with 1-, 2-, and 3-year RFS rates of 88.2%, 58.8%, and 58.8%, respectively. Median OS was likewise not reached, with corresponding 1-, 2-, and 3-year rates of 100%, 94.1%, and 70.3%. R0 resection was significantly associated with improved RFS (HR 0.20, 95% CI 0.04–1.01; log-rank P = 0.031) (**Table 5**).

**Table 5:** Comparison of key prognostic analyses between the overall cohort and the upfront-surgery subgroup.

Variable	Primary analysis (n = 20) HR (95% CI); *P*-value	Upfront-surgery subgroup analysis (n = 17) HR (95% CI); *P*-value
RFS		
R0 vs non-R0 resection	0.27 (0.07-1.10); *P* = 0.052	0.20 (0.04-1.01); *P* = 0.031
Radiotherapy vs no radiotherapy	0.34 (0.08-1.39); *P* = 0.117	0.29 (0.06-1.34); *P* = 0.091
OS		
Radiotherapy vs no radiotherapy	0.20 (0.03-1.23); *P* = 0.056	0.24 (0.03-1.80); *P* = 0.136
≥12 vs <12 chemotherapy cycles	–†; *P* = 0.030	–†; *P* = 0.052

†In both the primary analysis and the upfront-surgery subgroup analysis, no deaths occurred in the ≥12 chemotherapy cycles subgroup. Accordingly, Cox regression hazard ratio estimates cannot be calculated and only log-rank P values are reported. “–” denotes a hazard ratio that is not estimable.

HR, hazard ratio; CI, confidence interval; RFS, recurrence-free survival; OS, Overall survival.

## 4 Discussion

To our knowledge, the present study represents one of the most comprehensive adult-specific investigations of perioperative outcomes and prognostic factors in ssRMS, a rare and biologically heterogeneous entity for which dedicated adult-specific data have been largely absent. Among 20 adult patients receiving multimodality perioperative treatment, the mRFS was 37.9 months and the 3-year OS rate was 74.5%. These outcomes are encouraging relative to historical adult ssRMS series, yet remain substantially inferior to those reported in pediatric and congenital ssRMS populations. The key findings of this study are threefold: a non-significant directional association between R0 resection and improved RFS, a non-significant directional association between postoperative radiotherapy and OS, and a significant association between extended chemotherapy duration and OS, although the latter is limited by zero-event bias. Collectively, these observations suggest that treatment intensity and molecular subtype both contribute to outcome heterogeneity in adult ssRMS, and provide an empirical foundation for hypothesis-driven prospective investigation.

The survival outcomes observed in this cohort are best understood in comparison with both adult ssRMS series and broader RMS literature. Reported 5-year OS rates for embryonal RMS exceed 60%, while alveolar and pleomorphic subtypes carry substantially worse prognoses, a hierarchy that reflects well-established molecular and histological differences among subtypes (26). Within ssRMS specifically, congenital cases harboring VGLL2/NCOA2 fusions show 5-year survival rates approaching 91%, contrasting sharply with the considerably worse outcomes observed in adult ssRMS in both this and prior series (27). The 3-year OS rate of 74.5% in our cohort appears more favorable than outcomes reported in one of the largest prior ssRMS series, in which more than 50% of patients experienced recurrence or progression and only 7 of 16 (44%) remained disease-free survivors at a median follow-up of 39 months (18). Several factors may explain this difference. First, treatment intensity differed markedly: 40% of patients in the present cohort received ≥12 chemotherapy cycles, compared with only 38% receiving ≥6 cycles in the Japanese cohort, suggesting more aggressive systemic treatment. Second, postoperative radiotherapy was used in 70% of our patients versus 45% in Yasui et al., potentially contributing to better local control, particularly in non-R0 cases. Third, population differences cannot be excluded: the present cohort had a younger median age (31.5 vs. 45 years) and no retroperitoneal tumors (0% vs. 18%), both factors associated with relatively favorable prognosis in sarcoma literature (28, 29). However, these comparisons are exploratory and should not be interpreted as evidence of treatment superiority. Within ssRMS specifically, the recent multi-institutional series of 45 cases similarly identified head and neck primaries as the largest anatomic subset (40%, comparable to 50% in our cohort), supporting this region as a preferential site of ssRMS across geographically diverse populations (30).

Regarding surgical resection, the R0 resection group demonstrated a non-significant directional association with prolonged RFS that approached but did not reach statistical significance, likely reflecting insufficient statistical power in this small cohort. Nonetheless, the direction and magnitude of this effect are clinically meaningful and consistent with the recognized prognostic importance of surgical margins across RMS subtypes, where R0 resection is associated with 5-year survival rates of approximately 63% versus 46% for non-R0 patients (31). These data reinforce margin-negative resection as a central goal in ssRMS surgery. Whether neoadjuvant strategies could increase R0 rates and thereby improve outcomes in locally advanced ssRMS warrants dedicated investigation. This directional signal is concordant with the largest adult RMS series to date (n = 449), in which R0 resection emerged as an independent predictor of OS on multivariate analysis (32). Given that neoadjuvant therapy may influence resection outcomes, we further explored whether initial treatment strategy was associated with survival. An exploratory comparison between upfront surgery and neoadjuvant chemotherapy followed by surgery showed no significant differences in either RFS or OS. However, this analysis is limited by the very small number of patients receiving neoadjuvant chemotherapy and should be interpreted with caution.

Postoperative radiotherapy was administered to 70% of patients in this cohort. Although no statistically significant improvement in RFS was observed, there was a non-significant directional association toward improved OS in patients receiving radiotherapy versus those who did not. This pattern may suggest that radiotherapy contributes to survival through local control of subclinical disease rather than prevention of measurable progression events, a mechanism recognized in other high-grade soft tissue sarcomas (14). However, as radiotherapy was not randomly assigned, these comparisons are subject to treatment-selection bias and residual confounding. Although radiotherapy was recommended according to standard indications, final treatment decisions were influenced by patient preference and may correlate with unmeasured factors such as performance status or socioeconomic status. Notably, patients who declined radiotherapy were relatively enriched for R0 resection, indicating baseline imbalance in local-control status. Given the small sample size, formal adjustment for confounders was not feasible; therefore, the observed OS trend should be interpreted as exploratory. This directional finding is consistent with the NetSarc cohort, in which adjuvant radiotherapy was identified as an independent OS predictor in a larger, more heterogeneous population (32).

Before the 2021 NCCN and CSCO classifications formally designated ssRMS as a non-pleomorphic rhabdomyosarcoma subtype, chemotherapy regimens were selected based on rhabdomyosarcoma treatment principles and empiric regimens used in routine clinical practice. VAC became the institutional standard thereafter. Imbalances in three other areas further limit the valid interpretation of the comparison between the two chemotherapy regimens. Regarding baseline tumor characteristics, none of the patients in the VAC group had primary tumors exceeding 5 cm, whereas half of the patients in the non-VAC group had tumors larger than this size; this discrepancy reflects a difference in initial disease burden between the two groups. In terms of clinical staging, the VAC group consisted predominantly of patients with stage I disease (66.7%), whereas only 37.5% of patients in the non-VAC group were at stage I, with 50.0% presenting at stage III. This imbalance in staging reflects differences in baseline treatment selection, with a higher proportion of patients with advanced-stage disease in the non-VAC cohort. Regarding drug tolerability, the median number of chemotherapy cycles administered was 12 in the VAC group and only 6 in the non-VAC group. The lower number of cycles in the non-VAC group likely reflects the combined impact of greater baseline disease severity and increased surgical complexity, factors that may have limited the intensity of postoperative treatment. Accordingly, any comparison between regimens is exploratory and cannot be interpreted as evidence of equivalence or superiority.

Chemotherapy cycle number should be interpreted cautiously because completing ≥12 cycles required 9–12 months, making cycle count a post-treatment, time-dependent variable rather than a baseline assignment. This is especially relevant for RFS, as three patients in the <12-cycle group progressed at 7.1, 10.0, and 12.2 months, within or near the time needed to complete 12 cycles. Thus, immortal time bias and early-progression-related selection may have affected the RFS comparison, and chemotherapy duration should not be interpreted as causally reducing recurrence risk. For OS, immortal time bias appears less pronounced, as no deaths occurred during the 0–12-month treatment window and all five deaths in the <12-cycle group occurred at ≥16.4 months. However, selection bias remains substantial: patients completing ≥12 cycles may have had better performance status, treatment tolerance, lower perioperative morbidity, absence of early progression, or less aggressive tumor biology. Nevertheless, a biological rationale exists for extended chemotherapy: prolonged treatment may provide sustained suppression of micrometastases, consistent with the recognized tendency of ssRMS to retain chemosensitivity across multiple cycles (18, 33). Therefore, the observed association between chemotherapy duration and OS should be interpreted as potentially reflecting patient selection and underlying tumor biology rather than a direct therapeutic effect.

The exploratory molecular characterization in this cohort yielded several observations worth contextualization. Within the molecularly tested subset, progression and death events were observed only among patients harboring either MYOD1 mutations or ALK unbalanced translocations, whereas MYOD1 wild-type patients remained event-free during follow-up. Although the small subgroup sizes and non-uniform testing strategy preclude formal statistical inference, these findings suggest that these alterations may identify clinically aggressive subsets of adult ssRMS. The prognostic signal observed in MYOD1-mutant cases is directionally consistent with prior studies identifying MYOD1-mutant ssRMS as a high-risk entity irrespective of age, and with recent global characterization studies reaffirming MYOD1 mutation as a dominant alteration associated with aggressive adult ssRMS (8, 9, 34). The unfavorable clinical course among patients with ALK unbalanced translocations is broadly consistent with reports of aggressive spindle cell/epithelioid RMS associated with ALK upregulation and TFCP2 fusions (10, 35). However, because TFCP2 fusion status and ALK protein expression were not systematically assessed in this cohort, the therapeutic relevance of ALK-directed treatment remains uncertain. The absence of detected NCOA2 rearrangements is consistent with the view that VGLL2/NCOA2-rearranged ssRMS is predominantly an infantile or pediatric entity and uncommon in adults (36). These molecular observations should be interpreted cautiously because only 13 of 20 patients underwent molecular testing, the assays were not uniformly applied, and additional alterations beyond MYOD1, ALK, and NCOA2 were not systematically evaluated. Nevertheless, the clustering of adverse events among MYOD1-mutant and ALK-altered cases supports incorporating molecular profiling into the diagnostic workup of adult ssRMS for future risk stratification.

The subgroup analysis restricted to patients undergoing upfront surgery yielded findings consistent with those observed in the full cohort. Notably, the prognostic effect of R0 resection on RFS became statistically significant, whereas the associations of radiotherapy and chemotherapy duration with OS remained directionally unchanged. Together with the absence of survival differences between the upfront-surgery and neoadjuvant-treatment groups, these findings suggest that inclusion of the small neoadjuvant subgroup did not materially alter the principal conclusions, although the very limited sample size precludes definitive comparison between treatment strategies.

This study has several limitations that merit transparent acknowledgment. Inherent constraints of the single-center retrospective design and restricted sample size compromise statistical power, particularly for molecular subgroup analyses; all survival findings, especially those from zero-event subgroups, remain preliminary and require prospective validation. The events- per-variable criterion for multivariable Cox regression was not met (RFS: 9 events; OS: 5 events), precluding adjustment for confounders such as tumor size or molecular profile. Although exploratory comparisons between upfront surgery and neoadjuvant chemotherapy were performed, the very small number of neoadjuvant-treated patients precluded adequately powered comparisons and multivariable adjustment. Geographic restriction to a single center in Southwest China may also limit generalizability. Furthermore, the absence of external central pathology review represents an inherent limitation of single-center retrospective studies. However, all cases were systematically reassessed according to the 2020 WHO classification by a dedicated institutional sarcoma pathology team, which may address this concern and improve internal diagnostic consistency. Nonetheless, given the extreme rarity of adult ssRMS and the near- complete absence of prior perioperative outcome data specifically in adult patients, this study addresses a genuine and pressing evidence gap. The observations presented here, particularly regarding treatment intensity, radiotherapy use, and molecular heterogeneity, provide a structured empirical basis for the design of future multicenter prospective studies and international registry initiatives.

## 5 Conclusion

This study provides systematic perioperative outcome data in adult ssRMS, a rare entity for which dedicated adult evidence has been largely absent. Among 20 patients, the mRFS was 37.9 months and the 3-year OS rate was 74.5%, which is more favorable than prior adult sries yet substantially inferior to pediatric populations. Extended chemotherapy duration (≥12 cycles) was associated with improved OS, although this finding remains hypothesis-generating given the absence of deaths in the ≥12-cycle group. R0 resection showed a non-significant directional association with prolonged RFS, and postoperative radiotherapy showed a non-significant directional association with OS. MYOD1 mutations and ALK rearrangements identified subsets with higher progression and mortality rates, suggesting the potential value of molecular profiling for risk stratification. These results, derived from a single-center retrospective analysis with a restricted sample size, should be interpreted as preliminary and exploratory and require confirmation through large-scale, multicenter prospective investigations remains necessary.

## Data Availability

The original contributions presented in the study are included in the article/supplementary material. Further inquiries can be directed to the corresponding authors.
